# Health Education From 1775 to 2005

**Published:** 2005-10-15

**Authors:** Lynne S. Wilcox

Health education is an innate aspect of public health practice and difficult to discuss as a separate entity. Nevertheless, this special issue of *Preventing Chronic Disease* provides in-depth examinations of the purposes and uses of health education programs. We thank Neil Hann of the Oklahoma State Department of Health and Carol Russell of the Directors of Health Promotion and Education for serving as guest editors for this issue.

Among the earliest recorded health education programs in the United States were those related to military troops of the 18th century during the Revolutionary War. These programs are distinguished by their recognition of a "community," determined as much by membership in a common group as by geopolitical boundaries. Soldiers were more likely to die of infectious diseases than of battle wounds; camp hygiene was thus a critical aspect of an officer's duties. One of George Washington's first general orders, dated July 4, 1775, states, "All officers are required and expected to pay diligent Attention to keep their Men neat and clean . . . and inculcate upon them the necessity of cleanliness. . . . They are also to take care that Necessarys [latrines] be provided in the camps" (1).

Several programs discussed in this issue highlight health education in communities. A report from Texas describes the certification of *promotores* to serve as community health educators in neighborhoods (2). Oregon has developed a partnership between state public health and Medicaid agencies to encourage its community of health care providers to address the impact of tobacco on asthma morbidity (3). Colorado conducted an assessment of the costs and savings of community fluoridation programs within the state, providing useful information to policymakers on the importance of water fluoridation (4). North Carolina provided microgrants to empower local communities to select and implement their own health promotion projects (5), and in another program, encouraged local health departments to use policy-change and environmental-change strategies to address community risk factors (6).

One of the most remarkable reports on public health in the 19th century was the *Report of the Sanitary Commission of Massachusetts 1850*, also called the Shattuck Report after the chairman of the commission, Lemuel Shattuck (7). This document is considered the first scientific report in the United States describing the health of a population using birth and death rates, comparisons with the rates of other communities, and additional data to support its comprehensive recommendations on protecting the health of Massachusetts citizens.

One of the recommendations of the Shattuck Report addressed school health education: "Every thing connected with wealth, happiness and long life depends upon *health*. . . . This matter has been too little regarded in the education of the young. Intellectual culture has received too much and physical training too little attention. . . . By adopting [the recommendation], many and many a life would annually be saved in this Commonwealth, and the general health of the rising generation would be greatly improved" (7).

The health of school-aged and preschool-aged children receives noteworthy attention in this issue. Rhode Island surveyed school principals to assess current health promotion programs and then investigated the use of the School Health Index to improve school programs (8). Wisconsin established a resource guide for schools and families who care for children with diabetes (9). And Maine assessed the challenges of changing food options in school vending machines and cafeterias to improve student nutrition habits (10).

The Shattuck Report also recommended that "open spaces be reserved, in cities and villages, for public walks; that wide streets be laid out; and that both be ornamented with trees." The primary reason for this recommendation was to purify the air, but the report stated, "Open spaces also would afford to the artizan and the poorer classes the advantages of fresh air and exercise, in their occasional hours of leisure" (7).

In this issue, West Virginia describes a physical activity promotion project that encouraged schools, students, and communities to conduct small research programs in physical activity (11). Many of these emphasized walking routes and trails, providing the "fresh air and exercise" mentioned in the Shattuck Report.

Early in the 20th century, the Children's Bureau, a unit within the federal Labor Department, embarked on a massive media campaign, distributing 3 million pamphlets on infant care between 1914 and 1925 and responding to up to 125,000 letters each year from mothers (12). These communications extended to women of all races, classes, and regions, particularly poor rural women. One mother's letter noted, "Naturally I am much interested in the things being done for children. . . . In the course of a few years the Babies of today will be directing affairs."

Media campaigns continue to be an important aspect of health education. Oregon analyzed data from the Behavioral Risk Factor Surveillance System to identify whether at-risk Oregonians knew they were at high risk for developing diabetes (13). Knowledge gained from this survey will pave the way toward designing effective public health messages. Arkansas examined the effects of a radio campaign designed to increase physical activity among children aged 9 to 13 years (14).

In the 21st century, we continue to face similar challenges on health education, but we have new tools. Alabama describes an innovative approach to analyzing cancer data, which uses geocoding, a recently developed information tool, to identify unique population segments (15). In a collaborative partnership with state, federal, and private-sector members, the state linked information from the Behavioral Risk Factor Surveillance System, the U.S. census, health care use data, and marketing analyses of U.S. lifestyle segmentation clusters. The state cancer division added geocoding to 7 years of information from its cancer registry and used techniques developed by the National Cancer Institute's cluster-based Consumer Health Profiles. All these data will be used to identify Alabama's high-risk, underserved communities, develop and implement cancer programs designed for those communities, and assess the usefulness of such clustering approaches in cancer prevention and control among Alabama citizens.

Such a plethora of technical opportunities to collect and combine data was not available a decade ago. The multiple, unique programs presented in this issue illustrate the progress of U.S. health education over the past 230 years. While we have not yet achieved the goal of healthy lives for all, we have good reason to expect additional success in the future.
